# Secreted Wnt Modulators in Symptomatic Aortic Stenosis

**DOI:** 10.1161/JAHA.112.002261

**Published:** 2012-12-19

**Authors:** Erik Tandberg Askevold, Lars Gullestad, Svend Aakhus, Trine Ranheim, Theis Tønnessen, Ole G. Solberg, Pål Aukrust, Thor Ueland

**Affiliations:** Research Institute of Internal Medicine, Oslo University Hospital Rikshospitalet, Oslo, Norway (E.T.A., T.R., P.A., T.U.); Department of Cardiology, Oslo University Hospital Rikshospitalet, Oslo, Norway (E.T.A., L.G., S.A., O.G.S.); Section of Clinical Immunology and Infectious Diseases, Oslo University Hospital Rikshospitalet, Oslo, Norway (P.A.); Department of Cardiothoracic Surgery, Oslo University Hospital Ullevål, Oslo, Norway (T.T.); Center for Heart Failure Research, University of Oslo, Oslo, Norway (E.T.A., L.G., S.A., T.T.); Faculty of Medicine, University of Oslo, Oslo, Norway (L.G., T.R., T.T., P.A., T.U.)

**Keywords:** aortic stenosis, mortality, prognosis, Wnt

## Abstract

**Background:**

Valve calcification and inflammation play key roles in the development of aortic stenosis (AS). The Wnt pathways have been linked to inflammation, bone metabolism, angiogenesis, and heart valve formation. We hypothesized that soluble Wnt modulators may be dysregulated in symptomatic AS.

**Methods and Results:**

We measured circulating levels (n=136) and aortic valve tissue expression (n=16) of the secreted Wnt modulators secreted frizzled related protein-3, dickkopf-1 (DKK-1), and Wnt inhibitory factor-1 (WIF-1) by enzyme immunoassay, immunostaining, and RT-PCR in patients with symptomatic, severe AS and investigated associations with echocardiographic parameters of AS and cardiac function. Finally, we assessed the prognostic value of these Wnt modulators in relation to all-cause mortality (n=35) during long-term follow-up (median 4.6 years; survivors, 4.8 years; nonsurvivors, 1.9 years) in these patients. Our main findings were: (1) serum levels of all Wnt modulators were markedly elevated in patients with symptomatic AS (mean increase 231% to 278%, *P*<0.001), (2) all Wnt modulators were present in calcified aortic valves but correlated poorly with systemic levels or degree of AS, (3) some modulators (ie, WIF-1) were associated with the degree of myocardial function and valvular calcification, (4) all Wnt modulators, and DKK-1 in particular, predicted long-term mortality in these patients also after adjusting for conventional predictors including NT-proBNP.

**Conclusions:**

Together, these in vivo data support the involvement of Wnt signaling in the development of AS and suggest that circulating Wnt modulators should be further investigated as risk markers in larger AS populations, including patients with asymptomatic disease.

## Introduction

Calcification of the aortic valve is a slowly progressive disease continuum spanning from aortic sclerosis, with mild valve thickening, to marked calcification and impaired function of the calcific aortic valve in severe aortic stenosis (AS).^1^ The disease progression is now considered an active process and shares both risk factors and histopathologic features with atherosclerosis, including lipoprotein accumulation, inflammation, and remodeling of the extracellular matrix (ECM) with subsequent bone formation.^1–3^ Thus, infiltrating T cells and macrophages within the aortic valve may, at least partly, through release of cytokines, stimulate local production of matrix metalloproteinases (MMPs) in macrophages and valve interstitial cells that facilitate ECM remodeling and local calcification.^1,2^

The wingless (Wnt) proteins are a group of highly conserved secreted proteins of paramount importance in embryonic development. Through a complex interaction between different Wnt-pathways (ie, β-catenin-dependent [canonical] and β-catenin-independent [noncanonical] signaling), these proteins regulate cellular processes as diverse as proliferation, survival, cell fate determination, migration, and behavior.^4^ There is ample documentation for the importance of Wnt signaling in all aspects of normal heart development,^5,6^ including formation of the cardiac valves.^7^ The Wnt pathways have also been linked to inflammation, bone metabolism, angiogenesis, and matrix regulation, and altered Wnt signaling has been reported in a variety of disorders ranging from malignancies, osteoarthritis, and bone disorders to atherosclerosis.^4,8^ At present, however, there are limited data on the role of the Wnt system in the development of AS.^3^

The Wnt pathways are regulated by multiple families of secreted antagonists or modulators including secreted frizzled related proteins (sFRP) and dickkopfs (DKK) as well as Wnt inhibitory factor-1 (WIF-1*)*. Recent publications have demonstrated a role for WIF-1 as a regulator of osteoblastic differentiation^9^ and chondrogenesis^10^ in murine cells in vitro, thereby adding WIF-1 to the list of players in the network of Wnt signaling controlling skeletal development. The sFRP family of Wnt modulators has been linked to remodeling of the left ventricle (LV) following myocardial injury,^11^ and we have recently shown that DKK-1 may be involved in atherogenesis.^12^

On the basis of the role of Wnt signaling and its modulators in inflammation, LV remodeling, bone metabolism, and heart valve formation, we hypothesized that secreted Wnt modulators may be upregulated in symptomatic AS. In addition to examining serum levels of these proteins in patients with AS and in sex- and age-matched healthy controls, we examined their relationship to transvalvular gradients, valve area measures, and parameters of cardiac function, as well as their prognostic value in relation to all-cause mortality in these patients.

## Materials and Methods

### Patients and Controls

One-hundred thirty-six patients with symptomatic AS evaluated for aortic valve replacement (AVR) surgery were consecutively enrolled in the study as previously described.^13,14^ Characteristics of the patient group are given in Table 1. All patients underwent echocardiographic examinations and blood sampling, and all patients had confirmed AS assessed by echocardiography. Coronary angiography was performed in all patients to diagnose the presence of concomitant coronary artery disease (CAD; ie, at least 1-vessel disease [>50% narrowing of luminal diameter]). Patients with grade III aortic or mitral regurgitation or serum creatinine >150 μmol/L were not included in the study. All investigations were obtained within a few days' time. Of all 136 patients, 108 were scheduled for AVR surgery**,** whereas surgical intervention was declined in 28 (comorbidity/high risk for operation [n=19], unwilling to undergo operation [n=4], uncertain clinical benefit due to less severe symptoms [n=5]). Of patients accepted for AVR 2 died pending surgery, whereas 106 patients underwent surgical intervention. Serum from 95 healthy blood donors with no prior self-reported medical history and no regular use of any medication (mean±SD: 64±8 years, 42 women) was used for comparative analyses. The control subjects did not undergo a clinical examination, but height, weight, and smoking status were recorded, and blood was drawn for routine panel analyses. Baseline characteristics of patients and controls are given in Table 1. Associations of serum Wnt concentrations and aortic valve Wnt mRNA levels were investigated in 16 patients (mean±SD: 70±12 years, 7 women, 5 concomitant coronary artery bypass surgery) undergoing AVR surgery. Blood sampling was done 1 day preoperatively and aortic valve sampling as described below. Blood samples for the study were drawn from peripheral venous blood into pyrogen-free blood collection tubes without any additives and allowed to clot before centrifugation (1500*g* for 10 minutes). Serum samples were stored at −80°C and thawed <3 times.

**Table 1.** Baseline Characteristics of Patients and Controls

	n	Patients	n	Controls	*P*
Age, years	136	74.4 (9.1)	95	64.2 (7.9)	<0.001

Sex (male)	136	79 (58)	95	53 (55)	0.729

Smokers (never/ex/current)	131	68/18/45 (52/14/34)	94	57/6/31 (61/6/33)	0.358

BMI, kg/m^2^	131	26.3 (4.2)	95	25.1 (3.1)	0.024*

Hb, g/dL	135	13.5 (1.4)	95	14.1 (1.1)	0.006

WBC, 10^9^/L	135	7.6 (3.4)	95	5.6 (1.3)	<0.001

Platelets, 10^9^/L	132	229 (56)	95	275 (63)	<0.001*

HbA1c, %	94	5.7 (0.7)	95	5.5 (0.4)	0.334

eGFR, mL/min	136	72.8 (32.8)	92	91.2 (20.2)	<0.001

Total cholesterol, mmol/L	107	5.0 (1.1)	94	6.0 (0.9)	<0.001*

HDL cholesterol, mmol/L	132	1.6 (0.5)	93	1.8 (0.6)	0.043*

LDL cholesterol, mmol/L	127	3.2 (1.1)	93	3.8 (0.8)	<0.001*

NT-proBNP, pmol/L	136	244 (441)	93	9 (7)	<0.001

CRP, mg/L	136	5.5 (16.0)	77	2.0 (3.0)	0.002

sFRP3, ng/mL	136	1.99 (1.72)	92	0.86 (0.55)	<0.001

WIF-1, pg/mL	136	141.2 (103.3)	88	57.8 (106.8)	<0.001

DKK-1, ng/mL	135	4.14 (2.87)	92	1.49 (0.74)	<0.001

All variables displayed as mean (SD) or n (%) with corresponding *P* value.

* Student *t* test; all other variables assessed by the Mann–Whitney *U* test. BMI indicates body mass index; Hb, hemoglobin; WBC, white blood cell count; HbA1c, glycosylated hemoglobin fraction; eGFR, estimated glomerular filtration rate; HDL, high-density lipoprotein; LDL, low-density lipoprotein; NT-proBNP, amino-terminal pro B–type natriuretic peptide; CRP, C-reactive protein; sFRP3, secreted frizzled related protein 3; WIF-1, Wnt inhibitory factor 1; DKK-1, Dickkopf 1.

For serial measurements of secreted Wnt modulators, we included 22 patients with severe AS (mean aortic gradient >50 mm Hg or aortic area <0.7 cm^2^) who underwent AVR surgery as part of the ASSERT multicenter trial.^15^ Blood samples were drawn and echocardiographic measurements performed preoperatively, the second postoperative day, and at 6 and 12 months as previously described.^16^

All study subjects signed an informed consent form. The study protocol was approved by the Regional Committee for Medical and Health Research Ethics.

### Echocardiography

Continuous-wave Doppler from multiple positions was used to obtain the maximum aortic annular blood flow velocities and to calculate aortic valve area by use of the continuity equation.^17^ Doppler echocardiographic calculations of stroke volume and cardiac output were performed on the basis of the cross-sectional area of flow and aortic annular flow velocity data. Left ventricular ejection fraction (LVEF) was obtained by the biplane Simpson method.^18^ To obtain a semiquantitative measure of the morphology of the stenotic aortic valve, ultrasound backscatter data analysis was performed as previously described.^19^ Observers were blinded to patient clinical status and the standard echo findings.

### Biochemistry

N-terminal probrain natriuretic peptide (NT-proBNP) and C-reactive protein (CRP) were assayed on a MODULAR platform (Roche Diagnostics, Basel, Switzerland). High-sensitivity troponin T (hsTnT) was measured by electrochemiluminescence immunoassay (ELICA; Elecsys 2010 analyzer, Roche Diagnostics). Levels of WIF-1, sFRP-3, and DKK-1 were determined by enzyme immunoassay (EIA) provided by R&D Systems (Minneapolis, MN). Inter- and intraassay coefficient of variation (CV) was <10% for these assays. Plasma lipoprotein and creatinine levels were measured enzymatically on a Roche/Hitachi 917 analyzer (Roche Diagnostics, Mannheim, Germany). The estimated glomerular filtration rate (eGFR) was calculated using the Cockroft–Gault formula.

### Aortic Valve Sampling

Aortic valve specimens were obtained from patients (n=16) undergoing elective AVR surgery after signed informed consent. The valve cusps were excised as seen fit by the operating surgeon. Comparative immunohistochemical analyses were performed on aortic valves from persons with no medical history or macroscopic signs of heart disease (n=5) obtained from autopsies. Valve sampling was approved by the Regional Committee for Medical and Health Research Ethics. All aortic valve specimens were immediately immersed in formalin or liquid nitrogen where appropriate.

### Immunohistochemistry

Sections of paraffin-embedded aortic valves were treated with 0.5% H_2_O_2_, followed by high-temperature unmasking in citrate buffer (pH 6) blocked with 0.5% bovine serum albumin (BSA) and then incubated with primary antibodies against human sFRP-3 (polyclonal sc-13941, 1:400; Santa Cruz Biotechnology, Santa Cruz, CA), WIF-1 (monoclonal MAB134, 1:200; R&D Systems), and DKK-1 (polyclonal sc-25516, 1:200; Santa Cruz Biotechnology) for 1 hour at room temperature. After washing, the slides were incubated for 30 minutes with peroxidase-conjugated secondary antibodies (Impress-Vector, Vector Laboratories, Burlingame, CA), rinsed, and developed with chromogen for immunoperoxidase staining (DAB Plus, Vector laboratories) for 7 minutes. The sections were counterstained with hematoxylin.

### Quantification of Gene Expression

Total RNA was extracted from human aortic valve specimens using TRIzol (Invitrogen, San Diego, CA), DNase-treated, cleaned up using RNeasy Mini Columns (Qiagen, Hilden, Germany), and stored at −80°C until analysis. cDNA was made using a High Capacity cDNA Reverse Transcription Kit from Applied Biosystems (Foster City, CA). Quantification of gene expression was performed using an ABI Prism 7500 (Applied Biosystems), Power SYBR Green Master Mix (Applied Biosystems), and sequence-specific PCR primers (mRNA specific) designed using Primer Express software, version 3.0 (Applied Biosystems). Primer sequences can be provided on request. Gene expression of the housekeeping gene *GAPDH* was used as a reference for relative quantifications.

### Statistics

Because of nonnormal distributions of the investigated parameters, as assessed by the Shapiro–Wilk test, we used nonparametric statistics when analyzing differences between groups (eg, Mann–Whitney *U* test) and association between variables (Spearman correlation). The control population was ≍10 years younger than the patients (*P*<0.001), and the *P* values for differences between patients and controls have therefore been adjusted for age, sex, BMI, eGFR, and smoking status using linear regression (on log-transformed values of Wnt modulators). Kaplan–Meier analysis with log-rank test was performed to compare the number of events in relation to quartiles of the Wnt modulators (comparisons pooled over strata). Serial measurements of Wnt modulators in the longitudinal cohort were analyzed with the Friedman test. The importance of secreted Wnt modulators as risk factors for all-cause mortality was investigated by multivariable analyses. sFRP-3, WIF-1, and DKK-1 were included in separate models for the total population and in patients who underwent AVR in a Cox proportional hazard analysis (direct entry of covariates) including variables in Tables 1 and 2 that were imbalanced between survivors and nonsurvivors as a propensity score (ie, age, presence of type 2 diabetes mellitus, atrial fibrillation, eGFR, LVEF, aortic valve area, TnT, and NT-proBNP). The propensity score for each patient was made using binary logistic regression with population means replacing missing values. Non-Gaussian variables were log-transformed and adjusted for standard deviations. Harrell's C-index for survival analysis (ie, with censoring) was calculated for each model and is presented as area under the curve (AUC) with 95% CI. The assumption of proportional hazards was verified in all models using Schoenfeld residuals. Follow-up time for all-cause mortality was calculated from time of inclusion to death from any cause. *P* values are 2 sided and considered significant when <0.05. Statistical analyses were done using SPSS software (PASW version 18) and STATA version 11 for Windows (Stata Corp LP, Lakeway Drive, TX).

**Table 2.** Patient Characteristics and Association (Spearman Correlation, *r*) With Wnt Modulators in 136 Patients With Symptomatic AS

	Total Population	sFRP-3	WIF-1	DKK-1
Age, years	74±10	0.04	0.06	−0.13

Male, %	55	−0.09	−0.12	0.07

BMI, kg/m^2^	26.3±4.3	−0.10	−0.16	0.06

Coronary artery disease, %	43	0.05	0.11	0.02

Current smokers, %	33	−0.05	−0.06	0.03

DM, %	11	0.01	0.07	0.16

Hypertension, %	25	−0.17	−0.05	−0.12

Atrial fibrillation, %	34	0.15	0.13	0.15

Biochemistry				

HDL-Ch^†^, mmol/L	1.6 (1.3, 1.9)	−0.17*	−0.11	0.05

LDL-Ch^†^, mmol/L	3.0 (2.4, 3.9)	−0.11	−0.13	−0.09

eGFR^†^	66 (52, 86)	−0.13	−0.20*	0.19*

CRP^†^, mg/L	1.9 (0.9, 4.4)	0.26**	0.15	−0.08

hsTnT^†^, mmol/L	14.1 (8.3, 25.0)	0.01	0.23**	−0.12

Medication, %				

ACE inhibitors	14	−0.11	0.08	−0.10

ARB	19	0.04	−0.06	−0.03

Beta-blocker	45	−0.01	0.26**	−0.10

Statins	49	−0.05	0.12	−0.04

Warfarin	19	0.07	0.09	−0.09

Aspirin	47	−0.13	−0.03	−0.13

Values given as percentage, mean±SD, or ^†^median and interquartile range. BMI indicates body mass index; eGFR, estimated glomerular filtration rate; DM, diabetes mellitus; Ch, cholesterol; CRP, C-reactive protein; ACE, angiotensin-converting enzyme; ARB, angiotensin receptor blocker.

* *P*<0.05,

** *P*<0.01.

## Results

### Circulating Levels of Wnt Modulators in Patients With AS

**Figure 1.**
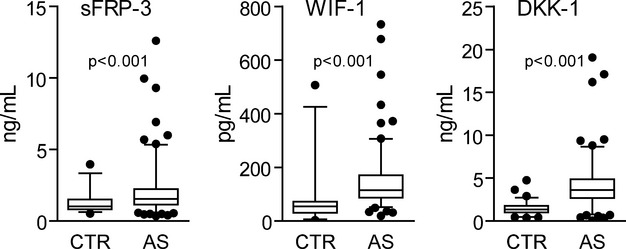
Serum levels of Wnt modulators in 136 patients with symptomatic aortic stenosis compared with healthy controls (n=95). Data are shown as a box and whisker plot with median (Q1, Q3) in the box and the whiskers representing the 5th and 95th percentiles. Outliers are shown as filled circles. *P* values are adjusted for age, sex, smoking status, BMI, and eGFR. AS indicates aortic stenosis; CTR, controls; BMI; body mass index; eGFR, estimated glomerular filtration rate.

Baseline characteristics of patients (n=136) and controls (n=95) are given in Table 1. Patients with symptomatic AS displayed markedly elevated serum levels of sFRP-3, WIF-1, and DKK-1 compared with healthy controls (Figure 1, Table 1). These differences persisted when adjusting for age, sex, BMI, eGFR, and smoking status (*P*<0.001 for all Wnt modulators) and when adjusting for all variables imbalanced between patients and controls (*P*<0.001 for all Wnt modulators). We found no significant correlations between the levels of secreted Wnt modulators and traditional risk factors for CAD (ie, smoking, lipids, hypertension, and obesity) except a positive correlation between HDL cholesterol and sFRP-3 (Table 2). In addition, elevated WIF-1 and decreased DKK-1 levels were seen in patients with a low eGFR (Table 2). In contrast to the lack of association with traditional risk markers, sFRP-3 was significantly correlated with CRP, and WIF-1 was significantly correlated with hsTnT, representing newer risk markers for CV disease (Table 2). Except for a significant association between elevated WIF-1 levels and the use of β-blockers, we found no associations between levels of Wnt modulators and the use of any particular medication (Table 2). Finally, when looking at correlations between the Wnt modulators, we found that sFRP-3 was positively correlated with WIF-1 (r=0.32, *P*<0.001) and DKK-1 (r=0.18, *P*=0.033).

### Levels of Secreted Wnt Modulators in Relation to Echocardiographic Measurements and Clinical and Neurohormonal Features of Myocardial Function

As can be seen in Table 3, WIF-1 and sFRP-3, but not DKK-1, were inversely correlated with LVEF. In contrast, no association between circulating Wnt modulators and aortic valve area or mean aortic gradient was observed. However, increased backscatter was seen in relation to elevated levels of WIF-1 (Table 3). Finally, a positive correlation was observed between neurohormonal activation (ie, NT-proBNP) and WIF-1, whereas none of the secreted Wnt modulators were associated with the degree of cardiac function, as measured by clinical assessment (ie, NYHA functional class).

**Table 3.** Echocardiographic Measurements and Clinical and Neurohormonal Features of HF in Relation (Spearman Correlation, *r*) to Levels of Wnt Modulators in 136 Patients With Symptomatic AS

	Total Population	sFRP3	WIF-1	DKK-1
NYHA functional class I/II/III/IV, %	4/40/75/1	−0.03	0.04	0.05

Hemodynamics				

LVEF, %	62±12	−0.18*	−0.23**	−0.12

CO^†^, mm Hg	4.8 (4.2, 5.6)	−0.08	−0.14	0.01

Aortic valve area^†^, cm^2^	0.62 (0.50, 0.80)	0.02	−0.13	−0.03

Mean aortic gradient, mm Hg	53.5±20.2	−0.13	0.01	−0.03

Backscatter, dB	18.8±5.0	−0.02	0.26**	0.12

Neurohormonal				

NT-proBNP^†^, pmol/L	98 (42 279)	0.02	0.27***	−0.08

Data are given as mean±SD or ^†^median (Q1, Q3). AS indicates aortic stenosis; sFRP, secreted frizzled related protein; WIF-1, Wnt inhibitory factor-1; DKK-1, dickkopfs-1; NYHA, New York Heart Association; LVEF, left ventricular ejection fraction; CO, cardiac output; and NT-proBNP, N-terminal probrain natriuretic peptide. To convert NT-proBNP values from p*M* to pg/mL, multiply by 8.47.

* *P*<0.05,

** *P*<0.01,

*** *P*<0.001.

### Presence of Wnt Modulators in Calcified Aortic Valves

**Figure 2.**
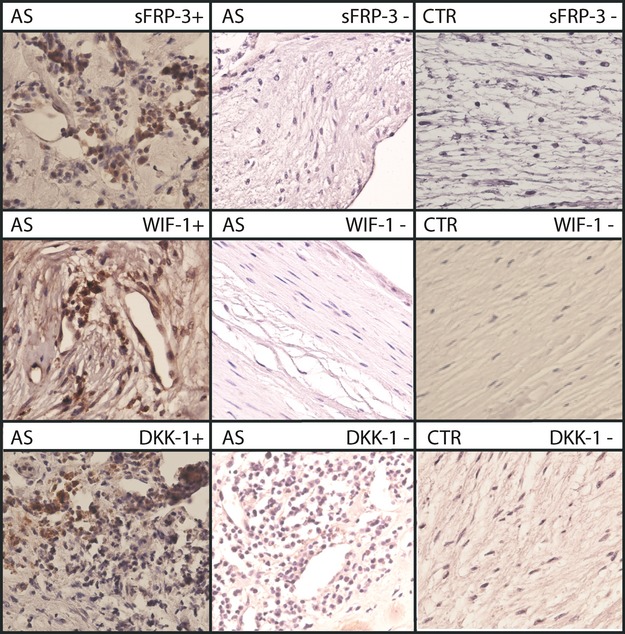
Immunohistochemical staining of sFRP-3, WIF-1, and DKK-1. Left panels show positive immunostaining of valves from patients with symptomatic AS. Middle and right panels show negative immunostaining of valves from patients with symptomatic AS and controls, respectively. Representative images obtained with ×40 objective. AS indicates aortic stenosis; sFRP, secreted frizzled related protein; WIF-1, Wnt inhibitory factor-1; DKK-1, dickkopfs-1; and CTR, controls.

To see if the Wnt modulators could be present in calcified aortic valves, we performed immunostaining of 6 aortic valves removed during AVR surgery, and immunohistochemistry revealed large heterogeneity in all valves studied, with varying degrees of positive staining. For sFRP-3, strong cell localization was observed in some individuals (Figure 2, left panel), whereas large areas displayed no staining (Figure 2, middle panel), with a similar pattern for DKK-1 and WIF-1 (Figure 2). Aortic valves from control patients did not reveal any staining with sFRP-3, WIF-1, or DKK-1 (Figure 2, right panel). Thus, although there was large heterogeneity, immunostaining suggested the presence of all these Wnt modulators within calcified aortic valves.

We next investigated whether the presence of Wnt modulators in the aortic valves could contribute to increased systemic concentrations by comparing sFRP-3, WIF-1, and DKK-1 mRNA levels in excised valves to paired preoperative serum samples in 16 patients. Although present in all samples, we found no correlations between systemic and local (ie, the aortic valve) sFRP-3, WIF-1, and DKK-1 levels (r=0.04, −0.09, and −0.03; *P*=0.88, 0.75, and 0.91, respectively), indicating no or only minor systemic effects of valvular-derived Wnt modulators.

### Effects of AVR Surgery on Systemic Levels of Wnt Modulators

**Figure 3.**
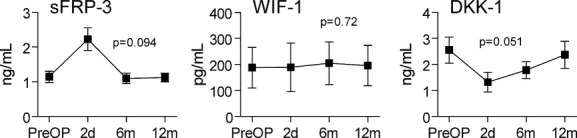
Circulating levels of sFRP-3, WIF-1, and DKK-1 in 22 patients undergoing AVR surgery. Data shown as mean±SEM. All Wnt modulators were measured preoperatively (PreOP), the second postoperative day (2d), and 6 months (6m) and 12 months (12m) after surgery. Corresponding *P* value for change over all times.

To further assess whether systemic Wnt modulator concentrations are influenced by the calcified aortic valve, we included a longitudinal cohort of 22 patients with severe AS who underwent AVR surgery. Although not significant (based on the a priori test), serial measurements (Figure 3) showed an increase in sFRP-3 concentration 2 days after AVR surgery, with return to preoperative levels after 6 months. An opposite pattern was seen for DKK-1, whereas WIF-1 showed only minor alterations in serum concentrations during follow-up.

### Circulating Wnt Modulators in Prediction of All-Cause Mortality

**Figure 4.**
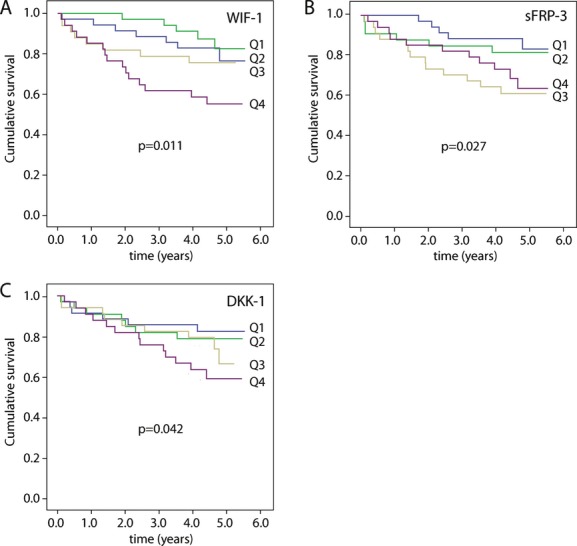
Wnt modulators in prediction of all-cause mortality. Kaplan–Meier plots comparing survival in relation to quartiles of the WIF-1 (A), sFRP-3 (B), and DKK-1 (C) with corresponding log-rank *P* values.

Finally, we investigated whether circulating Wnt modulator levels could predict all-cause mortality in our cross-sectional patient cohort (n=136). The patients were followed for a median (Q1 to Q3) duration of 4.6 years (4.0 to 4.9 years) in which 35 patients died, 13 in the nonsurgical group and 22 in the group that underwent surgery. Figure 4 shows Kaplan–Meier curves for associations between Wnt modulators (quartiles) and long-term mortality. Table 4 shows the association between levels of Wnt modulators (expressed per 1 SD change) and all-cause mortality in the total population and in patients undergoing AVR. Univariate analysis demonstrated an increased mortality rate in relation to increased levels of WIF-1 (HR 1.37, 95% CI 1.09 to 1.72, *P*=0.006)] and DKK-1 (HR 1.38, 95% CI 1.03 to 1.85, *P*=0.031), but not sFRP-3 (*P*=0.12). Furthermore, when adjusting for variables from Tables 1 and 2 that were imbalanced between survivors and nonsurvivors using a propensity score (see Statistics in Materials and Methods) in multivariable analysis (Table 4), increased levels of all Wnt modulators were associated with increased all-cause mortality: sFRP-3, HR 1.37, 95% CI 1.06 to 1.78, *P*=0.017; WIF-1, HR 1.44, 95% CI 1.05 to 1.99, *P*=0.025; DKK-1, HR 1.47, 95% CI 1.15 to 1.90, *P*=0.003. When patients who underwent AVR surgery were evaluated separately, DKK-1 was the strongest predictor (HR 1.67, 95% CI 1.29 to 2.18, *P*<0.001). However, receiver operating characteristic analysis indicated that the models had reasonable accuracy for the prediction of all-cause mortality at follow-up (Table 4).

**Table 4.** Circulating Wnt Modulators in Prediction of All-Cause Mortality

	sFRP-3		WIF-1		DKK-1	
			
	All Patients (n=32 deaths)	AVR (n=19 deaths)	All Patients (n=32 deaths)	AVR (n=19 deaths)	All Patients (n=32 deaths)	AVR (n=19 deaths)
Lg sFRP-3/SD	1.37 (1.06 to 1.78)	1.44 (1.11 to 1.87)				

Lg WIF-1/SD			1.44 (1.05 to 2.00)	1.42 (0.99 to 2.04)		

Lg DKK-1/SD					1.47 (1.15 to 1.90)	1.67 (1.29 to 2.18)

AUC*	0.83 (0.76 to 0.90)	0.81 (0.73 to 0.90)	0.83 (0.76 to 0.90)	0.80 (0.71 to 0.89)	0.84 (0.80 to 0.91)	0.85 (0.77 to 0.92)

Propensity score (PS) adjusted association between levels of Wnt modulators (expressed per 1 SD change) and all-cause mortality in the total population and in patients undergoing AVR. Data shown as HR (95% CI). AVR indicates aortic valve replacement; Lg, log-transformed variable; sFRP-3, secreted frizzle related protein 3; WIF, Wnt inhibitory factor; DKK-1, Dickopf 1.

* AUC calculated using Harrell's C-index, AUC (95% CI) with PS alone: all patients 0.82 (0.74 to 0.90), AVR 0.78 (0.68 to 0.88).

## Discussion

Our data showed that (1) serum levels of Wnt modulators were markedly elevated in patients with symptomatic AS, (2) all Wnt modulators were present in calcified aortic valves but correlated poorly with systemic levels or degree of AS, (3) some of these modulators (ie, WIF-1) were associated with the degree of myocardial function and valvular calcification, (4) Wnt modulators, and DKK-1 in particular, predicted long-term mortality in AS patients. Together, these in vivo data support the involvement of Wnt signaling in the development of AS and suggest that circulating Wnt modulators should be further investigated as risk markers in larger AS populations, including patients with asymptomatic disease.

We have recently demonstrated that serum osteoprotegerin^14^ and matrix gla-protein,^13^ key regulators of skeletal and vascular calcification, were markedly increased and associated with adverse outcome in symptomatic AS. Experimental models have demonstrated that low-density lipoprotein receptor-related protein 5, an important coreceptor in canonical Wnt signaling, regulates the expression of bone matrix proteins in the aortic valve and vasculature.^20^ Furthermore, Caira et al have suggested the involvement of Wnt signaling in the calcification of human aortic valves.^21^ Our study, showing markedly elevated serum levels of the Wnt modulators DKK-1, WIF-1, and sFRP-3 in patients with symptomatic AS associated with long-term mortality, supports the involvement of Wnt signaling in the development of AS. All the examined Wnt modulators were present in calcified aortic valves, suggesting they may regulate Wnt signaling in an autocrine/paracrine manner. However, the poor correlation between systemic and local (ie, aortic valve) Wnt levels or between systemic Wnt levels and degree of AS, both during cross-sectional and longitudinal testing, indicates that calcified valves are not a major source of circulating Wnt modulators.

The poor correlation with echocardiographic indices of AS could be explained by the homogeneity of our study population. All patients had symptomatic, severe AS, leaving a relatively narrow range of valve areas for analysis. Possibly, a study population ranging from aortic sclerosis to severe stenosis might reveal an association between circulating Wnt levels and echocardiographic parameters of AS. Furthermore, these Wnt modulators are widely expressed in many tissues (eg, vasculature and myocardium), and a concerted contribution from multiple tissues could explain the poor correlation between circulating levels of Wnt modulators and degree of AS.

Inflammation is an important component of atherosclerosis and vascular calcification,^1–3^ and the Wnt pathway may modulate the inflammatory response in this setting.^22^ For instance, experimental models indicate that bone loss during systemic inflammation in rheumatoid arthritis involves DKK-1.^23,24^ Furthermore, we have recently demonstrated a pathogenic role for DKK-1 in clinical atherosclerosis through enhancement of the inflammatory interaction between platelets and endothelial cells.^12^ Thus, the association between DKK-1 and adverse outcome in patients with symptomatic AS could reflect its ability to mirror inflammatory pathways. In our study, only sFPR-3 was correlated with CRP. However, although CRP may be a reliable marker of inflammation, it is unlikely that 1 biomarker could reflect all upstream pathways in a complex process like inflammation. Although calcification has been considered the predominant pathogenic process,^25^ inflammatory activity also contributes to the development of AS. Hence, it is possible that the association between circulating Wnt modulators and adverse outcomes in patients with symptomatic AS may reflect their ability to mirror both of these interacting processes.

The present study has some limitations. Relatively few patients were examined, undergoing relatively few events, and this lack of power has probably had an impact on our findings. Also, the control population was markedly younger, although this was accounted for in the statistical analysis, making it unlikely that the observed differences occurred by chance. The lack of data on patients with less severe AS, including aortic sclerosis, may have influenced the assumptions regarding associations between serum levels of Wnt modulators and degree of AS (ie, echocardiographic indices). Finally, the mixture of patients who underwent AVR and who did not may be a confounder.

To our knowledge, this study is the first to show that circulating Wnt modulators may be predictors of all-cause mortality in AS. Prospective studies, including both asymptomatic and symptomatic patients, as well as patients undergoing AVR, are needed to evaluate the prognostic relevance and clinical usefulness of elevated Wnt antagonists. Additional studies, with mechanistic emphasis, are needed to elucidate the pathogenic role of the Wnt signaling pathways in the development and progression of AS.
